# The Combined Use of Medium- and Short-Chain Fatty Acids Improves the Pregnancy Outcomes of Sows by Enhancing Ovarian Steroidogenesis and Endometrial Receptivity

**DOI:** 10.3390/nu14204405

**Published:** 2022-10-20

**Authors:** Xiangzhou Zeng, Siyu Li, Qianhong Ye, Shuang Cai, Shuang Quan, Lu Liu, Shihai Zhang, Fang Chen, Chuanjiang Cai, Fenglai Wang, Shiyan Qiao, Xiangfang Zeng

**Affiliations:** 1 State Key Laboratory of Animal Nutrition, Ministry of Agriculture Feed Industry Center, China Agricultural University, Beijing 100193, China; 2 Beijing Key Laboratory of Biofeed Additives, Beijing 100193, China; 3 State Key Laboratory of Agricultural Microbiology, College of Animal Sciences and Technology, Huazhong Agricultural University, Wuhan 430070, China; 4 Guangdong Provincial Key Laboratory of Animal Nutrition Control, College of Animal Science, South China Agricultural University, Guangzhou 510642, China; 5 College of Animal Science and Technology, Northwest A&F University, Xi’an 712100, China

**Keywords:** endometrial cells, granulosa cells, microbiome, reproduction, sodium butyrate, sodium caprylate, sodium laurate, sows

## Abstract

Fatty acids play important roles in maintaining ovarian steroidogenesis and endometrial receptivity. Porcine primary ovarian granulosa cells (PGCs) and endometrial epithelial cells (PEECs) were treated with or without medium- and short-chain fatty acids (MSFAs) for 24 h. The mRNA abundance of genes was detected by fluorescence quantitative PCR. The hormone levels in the PGCs supernatant and the rate of adhesion of porcine trophoblast cells (pTrs) to PEECs were measured. Sows were fed diets with or without MSFAs supplementation during early gestation. The fecal and vaginal microbiomes were identified using 16S sequencing. Reproductive performance was recorded at parturition. MSFAs increased the mRNA abundance of genes involved in steroidogenesis, luteinization in PGCs and endometrial receptivity in PEECs (*p* < 0.05). The estrogen level in the PGC supernatant and the rate of adhesion increased (*p* < 0.05). Dietary supplementation with MSFAs increased serum estrogen levels and the total number of live piglets per litter (*p* < 0.01). Moreover, MSFAs reduced the fecal Trueperella abundance and vaginal Escherichia-Shigella and Clostridium_sensu_stricto_1 abundance. These data revealed that MSFAs improved pregnancy outcomes in sows by enhancing ovarian steroidogenesis and endometrial receptivity while limiting the abundance of several intestinal and vaginal pathogens at early stages of pregnancy.

## 1. Introduction

In mammals, 30–50% of pregnancy loss occurs during early pregnancy, profoundly influenced by embryo implantation [1]. Successful embryo implantation requires proper embryo development and a receptive endometrium. Maintaining ovarian function has great significance for both embryo development and endometrial receptivity. Ovarian granulosa cells synthesize various hormones and growth factors to regulate the growth, differentiation and maturation of follicular membrane cells and oocytes, as well as follicular development [2]. The reproductive hormones produced by ovarian granulosa cells also play important roles in ensuring the development of embryos and endometrial receptivity [3,4,5,6]. Meanwhile, the endometrium goes through a series of cyclic changes and remains receptive for a short time during early pregnancy [7].

Lipid metabolism plays significant roles in embryo development and implantation by converting lipids to lysophosphatidic acid, cannabinoids, prostaglandins, sphingosine-1-phosphate and steroid hormones [8,9,10,11,12]. As important metabolites of lipids, fatty acids play critical roles in embryonic development and implantation [13,14,15]. In vitro experiments showed that adding 1 μM docosahexaenoic acid (DHA) to the medium improved the quality of bovine oocytes, embryo development capacity and blastocyst cell number, while arachidonic acid treatment increased plasma phospholipase A-2α (CPA-2 α) phosphorylation in mouse endometrial stromal cells. Additionally, arachidonic acid could induce prostaglandin-endoperoxide synthase 2 (PTGS2) expression in endometrial interstitial cells and then promote embryo implantation and decidualization [16]. Similarly, in vivo studies found that conjugated linoleic acid rich diet reduced the expression of cannabinoid receptor 2 (CNR2) and increased the progesterone content in cattle [17]. However, long-chain saturated fatty acids often have a negative effect on reproductive performance. Nonesterified fatty acids reduce the bovine blastocyst development rate and total cell number in vitro [18]. In vitro cultures of mouse embryos in palmitic acid increase the mRNA abundance of insulin-like growth factor 1 (IGF-1), and glutamate pyruvate transaminase (GPT2) reduces the total cell number of blastocysts and increases the apoptotic rate of trophoblast cells [19]. In contrast, in addition to providing energy for cells, short- and medium-chain fatty acids exhibit various regulatory and signaling functions [20,21,22]. However, there are only a few reports on the effects of medium- and short-chain fatty acids on reproductive performance. In a study of rats, diets were supplemented with either high fiber or butyric acid (SB), and both increased fetal number and reduced miscarriage rates. Among them, a high-fiber diet enhanced the antioxidant capacity in maternal rats and fetuses, while sodium butyrate had no effect on antioxidant capacity, demonstrating that the mechanism by which sodium butyrate promotes reproductive performance may be different from that of a high-fiber diet, but the specific mechanism is still unknown [23]. By measuring the metabolites (mainly amino acids and fatty acids) and labeling caprylic acid in the culture medium of early embryos of mice at different stages, it was found that caprylic acid could provide energy for the early mouse embryos. In particular, in the absence of energy substances in the culture medium, the early mouse embryos consumed more caprylic acid [24]. As mentioned above, research has shown that various fatty acids have impacts on the development of early pregnancy, but studies on the reproductive performance of short- and medium-chain fatty acids are relatively limited, and the related mechanism is not clear. In addition, the effect of the combined use of different fatty acids on reproductive performance is lacked.

Our previous study demonstrated that the supplementation of short- and medium-chain fatty acids in rat diet could improve embryo implantation [25]. However, the effects of a combination of short- and medium-chain fatty acids on the function of granulosa cells and endometrial cells in early pregnancy are unknown, as are the effects on intestinal and vaginal microbial composition in early pregnancy. The aim of this study was to investigate whether a rational combination of functional short- and medium-chain fatty acids could provide a new nutritional strategy for ensuring successful pregnancy by improving the function of gestational ovarian granulosa cells and endometrial cells while regulating the microbiological environment of the intestine and vagina in early pregnancy, thus providing stable hormone levels and a favorable attachment environment in early pregnancy.

## 2. Materials and Methods

### 2.1. Porcine Primary Cultured Ovarian Granulosa Cells

Porcine ovaries were purchased from a Hebei slaughterhouse (China) and transported to the laboratory at 37 °C in NaCl (0.9%) within 30 min. Ovaries (n = 60) were rinsed at 37 °C in 0.9% NaCl with 2% penicillin–streptomycin solution (0.1 mg/mL streptomycin + 10 mg/mL penicillin) (Gibco) until no blood was visible. The follicles with an estimated diameter greater than 5 mm (visually measured) were opened with a 5 mm syringe and 20-G needle (sterile). The follicular fluid (FF) and cumulus-oocyte complexes (COCs) were transferred to several sterile centrifuge tubes (15 mL) and then centrifuged at 200× *g* for 5 min, followed by the removal of the remnant supernatant. After that, 5 mL of PBS containing 10% penicillin–streptomycin solution at 37 °C was added to the centrifuge tubes, and the pellet was carefully resuspended and cleaned. Granulosa cells (GCs) were extracted through centrifugation (200× *g*, 5 min), then seeded onto culture dishes. Dulbecco’s modified Eagle’s medium (DMEM) (high glucose) (Sigma–Aldrich, St. Louis, MO, USA), fetal bovine serum (FBS) (Gibco, Carlsbad, CA, USA), 10 mg/mL penicillin and 0.1 mg/mL streptomycin were the components in the culture medium used. Cell cultivation was conducted at 37 °C under 5% CO_2_ aerobic conditions for 3 h, and then the medium was replaced with fresh culture medium. Next, the cells were cultured for 2 days and treated with 1 mM SB (sodium butyrate, Sigma-Aldrich), 0.04 mM SC (sodium caprylate, Sigma-Aldrich), 0.01 mM SL (sodium laurate, Sigma-Aldrich) and a combination of all three (MSFAs,1 mM SB + 0.04 mM SC + 0.01 mM SL) for 24 h; the optimal concentrations of these three fatty acids were obtained with a CCK-8 kit. The experiment was repeated three times, each with three parallels.

### 2.2. Cell Viability Assay

With different concentrations of SB (0, 0.1, 0.4, 0.6, 1.0 and 1.4 mM) and SC (0, 0.2, 0.4, 0.6, 0.8 and 1.0 mM), and SL (0, 0.005, 0.008, 0.01, 0.05 and 0.1 mM) treated PGCs for 24 h. The cell activity of PGCs under different treatments was detected according to the instructions of CCK-8 kit (Abcam, Shanghai, China). After testing, we found that 1.0 mM SB, 0.4 mM SC, 0.01 mM SL was the optimum concentration (Figure S1) and used for subsequent cell assays.

### 2.3. Sex Hormone and Blood Biochemistry Detection

Commercially available radioimmunoassay kits (Cusabio) (Beijing North Institute of Biotechnology, Beijing, China) were used to detect the progesterone and estradiol concentrations in PCG culture supernatants. Total cholesterol, HDL cholesterol and LDL cholesterol were measured using kits from Huaying Institute of Biotechnology (Beijing, China).

### 2.4. Cell Culture

Porcine endometrial epithelial cells (PEECs) were used to study endometrial receptivity and cultured in medium [90% Dulbecco’s modified Eagle’s medium (DMEM) (high glucose) and 10% FBS (Gibco)] and conducted at 37 °C in a humidified 5% CO_2_ incubator. Cells serum was starved before treatment. The cells were also treated with 1 mM SB, 0.04 mM SC, 0.01 mM SL and MSFAs for 24 h, and the experiment was repeated three times, each with three parallels. PEECs is a cell line that was kindly provided by Dr. Guoyao Wu from China Agricultural University.

Dulbecco′s Modified Eagle′s Medium/Nutrient Mixture F-12 Ham (DMEM/f-12) supplemented with 10% FBS (Gibco) and 1% insulin, transferrin and selenium solution (ITS-G) (Gibco) was used to culture the porcine trophoblast cells (pTrs), and the cells were maintained at 37 °C in a humidified 5% CO_2_ incubator. The pTr cells were purchased from the European Collection of Cell Cultures.

### 2.5. qPCR Analysis

Quantitative PCR (qPCR) was performed according to previously described methods [25]. Primers were designed using Primer 3.0 plus input online (Table 1).

### 2.6. Adhesion Assay

PEECs (1.5 × 10^6^ cells) were cultured in 6-well plates for 24 h. The medium was replaced and incubated in DMEM (high glucose) with different fatty acids (1 mM SB, 0.4 mM SC, 0.01 mM SL, MSFAs) for 24 h. pTr cells were labeled with fluorescent dye 5-chloromethylfluoresceindiacetate (CMFDA) (CellTracker Green; Invitrogen, Carlsbad, CA, USA) for 10 min at 37 °C. Then, the labeled pTr cells were cleaned in 1 × phosphate-buffered saline and incubated with PEECs for 30 min at 37 °C. The cells were vigorously washed to remove nonattached pTr cells. The attached pTr cells were observed with a fluorescence microscope (Axioimager M1 microscope, Zeiss, Aalen, Germany), and the cell adhesion rates were calculated [26].

### 2.7. Animals and Experimental Design

The animal procedures described in this experiment were approved by the Institutional Animal Care and Use Committee of China Agricultural University (CAU 20161108-2), and animal experiments were finished in the FengNing Swine Research Unit of China Agricultural University (Academician Workstation in Chengdejiuyun) Agricultural and Livestock Co., Ltd., Hebei, China.

To confirm whether maternal fatty acid supplementation during early pregnancy improved reproduction in sows, among 60 Large White × Landrace crossbred sows with similar body weight (BW) and backfat thickness (BF) were used in this study. After weaning, sows were randomly allocated into two groups based on BW, BF and parity. Sows were feed singly in gestation stalls (2.2 m × 0.65 m); using direct boar exposure to check daily for estrus in the morning, artificial insemination was performed during estrus (12 h apart). Sows were not used in this experiment if they returned to estrus after insemination. In the control group, sows were fed basal diets from the time of weaning to the early pregnancy period (days 0 to 28 of gestation). In the treatment group, sows were fed the basal diet supplemented with 0.1% sodium butyrate, 0.04% sodium caprylate, and 0.1% sodium laurate (purity > 98%, Newgrun Biotechnology, Changsha, China). After day 28 of gestation, all groups were fed the same basal diet. All sows were fed 2.00 kg of the diet during weaning to the end of breeding, 1.25 kg of the diet during early pregnancy and 1.75 kg of the diet from day 29 to delivery and fed twice daily at 06:30 h and 16:30 h. Sows drank water freely throughout the experiment. Diets met the nutritional requirements of pregnant sows (GB/T 39235-2020 Nutritional Requirements for Swine) (Table 2), and the dietary information for diets with fat supplementation is showed in Table S1. Sows’ body weight and back fat were measured on days 0 and 28 of gestation. On days 14 and 28 of gestation, after cleaning, microbes from the vagina and rectum of sows were collected with sterile cotton swabs (Baiao Biological Technology, Shenzhen, China) and immediately frozen in liquid nitrogen. On day 28 of pregnancy, after overnight starvation, 10 sows were randomly selected from each group for blood sample collection via jugular venipuncture into 5 mL tubes, and the samples were centrifuged at 3000× *g* for 10 min at 4 °C to obtain serum samples. Shortly after birth, litter size, live litter size, litter weight, number of mummified and stillborn piglets and birth weight were recorded.

### 2.8. Bacterial DNA Extraction and 16S rRNA Gene Sequencing

Microbial community genomic DNA was extracted from vaginal and rectal swabs using the QIAmp DNA stool mini kit (Qiagen, GmbH, Hilden, Germany). The DNA extract was checked by agarose gel electrophoresis, and DNA concentration and purity were detected by NanoDrop 2000 UV–vis spectrophotometer (Thermo Scientific, Wilmington, NC, USA). The PCR primers flanking the V3-V4 of the bacterial 16S rDNA gene were amplified with primer 338F (5′-ACTCCTACGGGAGGCAGCAG-3′), and the reverse primer was 806R (5′-GGACTACHVGGGTWTCTAAT-3′) using an ABI GeneAmp^®^ 9700 PCR thermocycler (ABI, Vernon, CA, USA). The optimized conditions for PCR amplification of the 16S rRNA gene were follows: 1 pre-denaturation cycle at 95 °C for 3 min, then 27 cycles of denaturing at 95 °C for 30 s, annealing at 55 °C for 30 s, elongation at 72 °C for 45 s and a single extension at 72 °C for 10 min, ending at 4 °C. Following the standard protocols by Majorbio Bio-Pharm Technology Co., Ltd. (Shanghai, China), the purified amplicons were pooled in equimolar amounts and paired-end sequenced on an Illumina MiSeq PE300 platform/NovaSeq PE250 platform (Illumina, San Diego, CA, USA); fastp version 0.20.0 [27] was used to demultiplexed raw 16S rRNA gene sequencing reads, quality filtered by and merged in FLASH version 1.2.7 [28] with the following criteria: (i) the 300 bp reads were truncated at any site receiving an average quality score of <20 over a 50 bp sliding window, and the truncated reads shorter than 50 bp were discarded. 

Operational taxonomic units (OTUs) with a 97% similarity cutoff [29,30] were clustered using UPARSE version 7.1 [29], and chimeric sequences were identified and removed. The taxonomy of each OTU representative sequence was analyzed in RDP Classifier version 2.2 [31] against the 16S rRNA database (Silva v138) using a confidence threshold of 0.7. We performed α- and β-diversity calculations and taxonomic community assessments using Qiime2-2020.11 scripts. The alpha diversity of the samples was calculated with the observed species, Chao 1 and Shannon indexes. Principal coordinate analysis (PCoA) based on the weighted UniFrac distance was used to summarize the beta diversity. Linear discriminant analysis coupled with effect size (LEfSe) was used to identify the key bacterial taxa between different treatments [32].

### 2.9. Statistical Analysis

The results are expressed as the mean ± standard error of the mean (SEM). Statistical analysis was performed using a nonpaired Student’s t test or one- or two-way ANOVA in Statistical Product and Service Solutions (SPSS) software (version 26.0.0, IBM, Armonk, NY, USA). Tukey’s post hoc test was applied to determine the differences among treatment means, and a probability of *p* ≤ 0.05 was considered significant. Linear discriminant analysis (LDA) effect size (LEfSe) was calculated in R 4.0 to identify enriched OTUs, the threshold of the logarithmic score of LDA was set to 2.0, and *p* ≤ 0.05 and LDA > 2.0 were considered significant across different stages of gestation. The data were visualized using R 4.0 and GraphPad Prism 8.1 (GraphPad Software, San Diego, CA, USA).

## 3. Results

### 3.1. MSFAs Can Increase the Expression Levels of Steroid Steroidogenesis Genes and Luteinizing Genes and Promote Estrogen Synthesis

**Figure 1:**
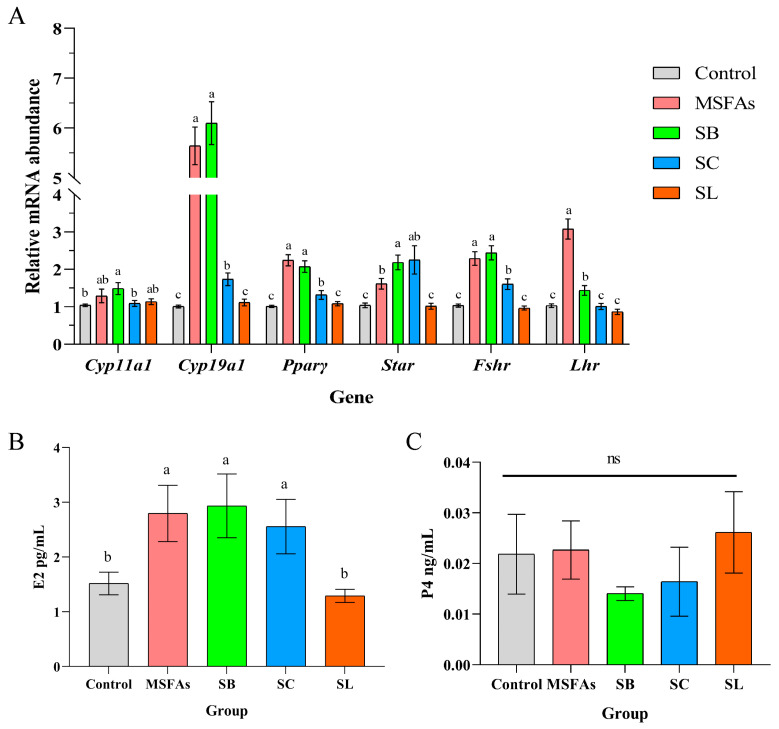
MSFAs can increase the expression levels of steroid steroidogenesis genes and luteinizing genes and promote estrogen synthesis. (**A**) The relative mRNA abundance in porcine ovarian granulosa cells treated with 1 mM SB, 0.4 mM SC, 0.01 mM SL or their combination for 24 h. (**B**) Concentrations of progesterone and (**C**) estradiol in the supernatant of porcine ovarian granulosa cells treated with different short-chain fatty acids, medium-chain fatty acids or their combination for 24 h. 1 mM SB; 0.4 mM SC; 0.01 mM SL. The results are presented as the means ± SEM. n = 9. Different letters between bars indicate *p* ≤ 0.05 by one-way ANOVA followed by post hoc Tukey’s tests. ns, nonsignificant (*p* > 0.05). SB, sodium butyrate; SC, sodium caprylate; SL, sodium laurate; MSFAs, SB + SC + SL.

As shown in Figure 1A, the relative mRNA abundance of cytochrome P450 family 19 subfamily A member 1 (*Cyp19a1*), follicle-stimulating hormone receptor (*Fshr*), luteinizing hormone receptor (*Lhr*), peroxisome proliferator activated receptor gamma (*Pparγ*) and steroidogenic acute regulatory protein (*Star*) were higher in the MSFA, SB and SC groups than in the control and SL groups. In the SB group, the relative mRNA abundance of *Cyp11a1* was higher than that in the control group and SC group, but there was no significant difference compared with the abundance in the MSFA and SL groups. Compared with the SC group, the relative mRNA abundance of *Cyp19a1*, *Pparγ*, and *Fshr* was higher in the MSFA and SB groups. However, in the MSFA group, the relative mRNA abundance of *Lhr* was higher than that in the other groups.

Based on the changes in the relative mRNA abundance of the genes related to hormone synthesis in cells, we examined the progesterone and estradiol concentrations in the supernatant and found that there was no significant influence on progesterone concentrations in any group but that the MSFA, SB and SC groups showed significant improvement in the cell supernatant concentrations of estradiol, which was consistent with the qPCR results (Figure 1B,C). Taken together, our results demonstrated that MSFAs could promote steroidogenesis and the expression of LH and FSH receptor-related genes and enhance the secretion of estrogen in PGCs.

### 3.2. MSFAs Can Improve the Receptivity of Pig Endometrial Cells

**Figure 2:**
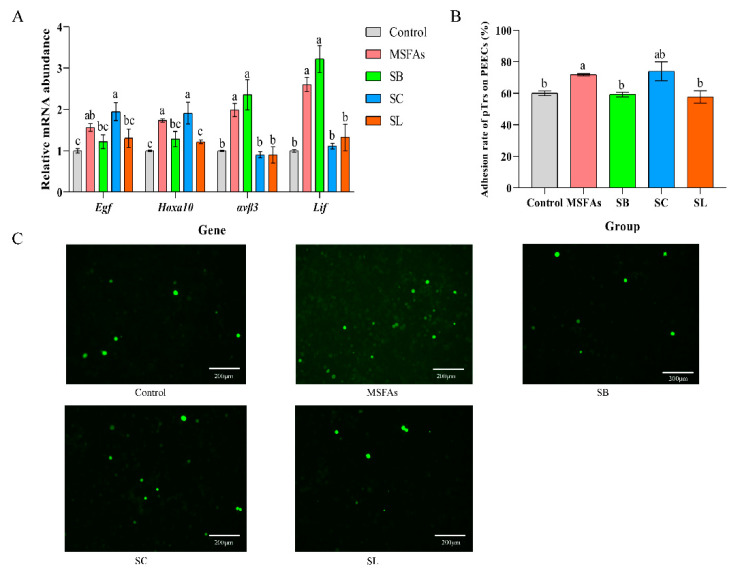
The combination of MSFAs can improve the receptivity of pig endometrial cells. (**A**) The relative mRNA abundance in porcine endometrial epithelial cells treated with 1 mM SB, 0.4 mM SC, 0.01 mM SL or their combination for 24 h. (**B**) Adhesion rate of porcine trophoblast cells to porcine endometrial epithelial cells treated with 1 mM SB, 0.4 mM SC, 0.01 mM SL or their combination for 24 h. (**C**) Image of adhesion of porcine trophoblast cells to porcine endometrial epithelial cells treated with 1 mM SB, 0.4 mM SC, 0.01 mM SL or their combination for 24 h observed under fluorescence microscope. The results are presented as the means ± SEM. n = 9. Different letters between bars indicate *p* ≤ 0.05 by one-way ANOVA followed by post hoc Tukey’s tests. ns, nonsignificant (*p* > 0.05). SB, sodium butyrate; SC, sodium caprylate; SL, sodium laurate; MSFAs, SB + SC + SL.

Improvement in the receptivity of pig endometrial cells is beneficial to early embryo implantation and can effectively improve the reproductive performance of sows. We measured the relative mRNA abundance of endometrial receptivity genes in porcine endometrial cells treated with different short-chain fatty acids and their combinations. As shown in Figure 2A, compared with the control group, MSFA supplementation significantly improved the relative mRNA expression levels of epidermal growth factor (*Egf*), homeobox A10 (*Hoxa10*), integrin subunit beta 3 (*ανβ3*) and leukemia inhibitory factor (*Lif*). SB supplementation improved the expression of *ανβ3* and *Lif*, SC treatment improved the expression of *Egf* and *Hoxa10*, and SL treatment only improved the expression of *Hoxa10*. There were no differences in the relative mRNA abundance of *Egf* in the MSFAs, SB and SL groups, but the expression in the SC group was higher than that in the SB and SL groups. In both the MSFAs and SC groups, the expression of *Hoxa10* was higher than that in the other groups. However, the expression of *ανβ3* and *Lif* in the MSFA and SB groups was significantly increased than that in the other groups.

As shown in Figure 2B, the combination of medium- and short-chain fatty acids improved the rate of adhesion of trophoblast cells to endometrial cells, which was consistent with the RT–PCR results. Collectively, these results indicated that MSFAs can improve the receptivity of endometrial cells in sows.

### 3.3. Effects of Dietary Supplementation with MSFAs on the Reproductive Performance of Sows

As shown in Table 3, four sows in both the control and treatment groups failed to fertilize during pregnancy; additionally, dietary supplementation of MSFAs in the estrus and early gestation had no effect on the body weight and backfat thickness of sows (*p* > 0.05). Dietary supplementation of short-chain fatty acids in the estrus and early gestation of sows tended to increase the total litter size (*p* = 0.078). Compared with that in the control group, the number of live litters born in the treatment group was significantly increased (*p* < 0.01), and there was also an increasing trend observed for the litter weights of live litters (*p* = 0.09). Treatment had no effect on average birth weight, sex, offspring strength, stillbirth occurrence, deformation or mummification (*p* > 0.05) (Table 4).

### 3.4. Effects of Dietary Supplementation of MSFAs on Serum Steroid Hormones and Serum Metabolites at Day 28 of Gestation

As shown in Table 5, dietary supplementation with the medium-/short-chain fatty acid combination in the treatment group significantly increased the estradiol content in the serum of sows on day 28 of gestation (*p* < 0.05) but had no effect on cholesterol synthesis related to serum lipid metabolism in the sows (*p* > 0.05).

### 3.5. Effects of Dietary Supplementation with MSFAs on the Rectal Swab Microbial Diversity and Composition of Sows at 14 and 28 Days of Gestation

**Figure 3:**
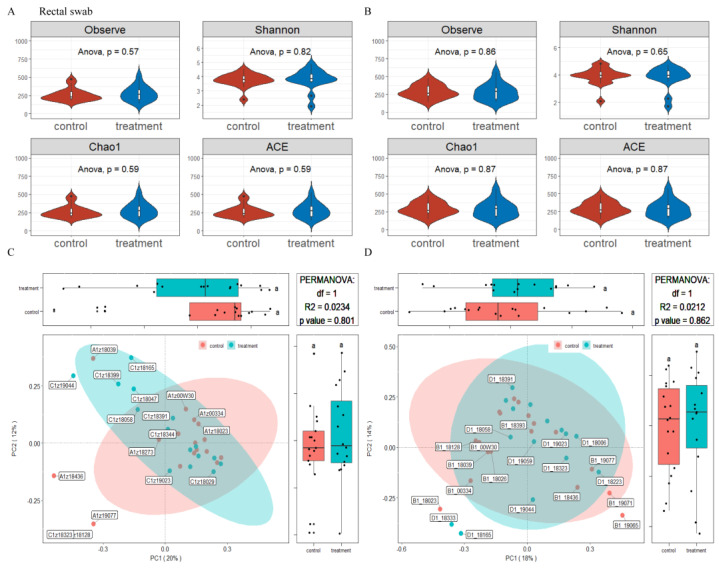
Effects of MSFAs during gestation on the rectal swab microbiota diversity of sows. Comparison of the number of observed species and Shannon, Chao1 and ACE indexes between the control and treatment groups at day 14 (**A**) and day 28 (**B**) of gestation. Beta diversity of the fecal microbiota in sows between the control and treatment groups at day 14 (**C**) and day 28 (**D**) of gestation based on weighted UniFrac distance. Data are expressed as the mean ± SEM (*n* = 11).

The microbiota diversity and compositions of rectal swab samples in sows at 14 and 28 days of gestation were determined by deep sequencing the 16S rRNA genes. To assess rectal swab microbial community structure, we calculated the alpha and beta diversity values (Figure 3). For richness indexes (observed species, Chao1), dietary supplementation with MSFAs had no effect (*p* > 0.05). On the other hand, beta diversity is shown in Figure 3. PCoA clustering revealed that the rectal swab microbiota of sows was not significantly dispersed in the control and treatment groups (*p* > 0.05). In conclusion, the addition of MSFAs to the diet did not effectively change the rectal swab microbiota diversity of sows at 14 or 28 days of gestation.

**Figure 4:**
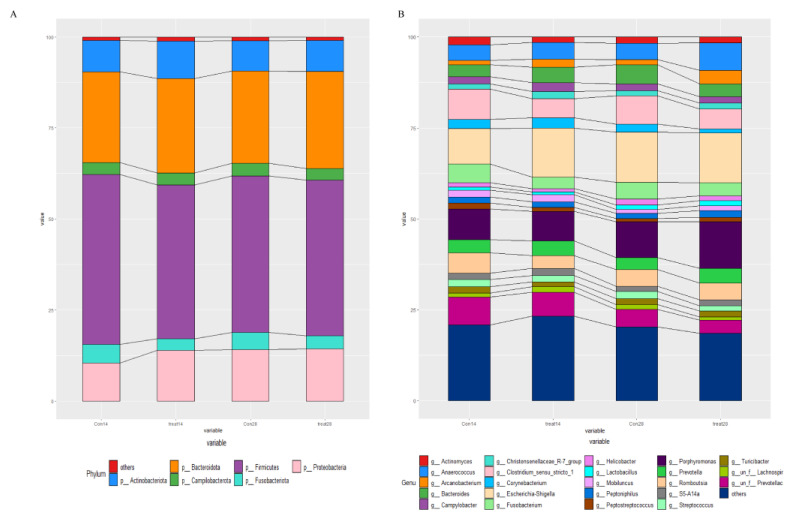
Effects of MSFAs during gestation on the rectal swab microbiota compositions of sows. The relative abundances of different phyla (**A**) and genera (**B**) in the rectal swab microbiota of control and treatment group sows at day 14 and day 28 of gestation. *N* = 11.

**Figure 5:**
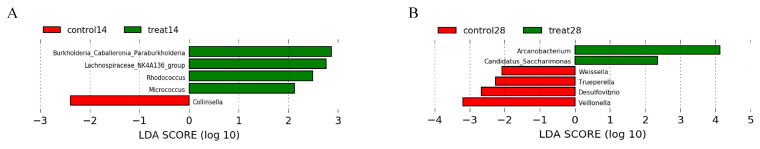
Effects of dietary supplementation with MSFAs on the rectal swab microbial diversity and compositions of sows at 14 and 28 days of gestation. Linear discriminant analysis coupled with effect size (Lefse) of day 14 (**A**) and day 28 (**B**) rectal swab microbiota compositions in the control and treated sows. Control14 (28), day 14 (28) of gestation and fed the basal diet. Treat14 (28), day 14 (28) of gestation and fed a basal diet supplemented with 0.1 SB, 0.05 SC and 0.1% SL.

The relative abundances at the phylum and genus levels in the rectal swab samples of all groups across different days are shown in Figure 4. The top six phyla were *Firmicutes*, *Bacteroidota*, *Proteobacteria*, *Actinobacteriota*, *Fusobacteriota* and *Campylobacterota* (Figure 4A). These six phyla accounted for more than 99% of the reads for all sows. In addition, the top six genera in the gut microbiota of sows were *Escherichia-Shigella*, *Clostridium_sensu_stricto_1*, *Porphyromonas*, *Prevotellaceae*, *Anaerococcus* and *Romboutsia* (Figure 4B). The microbial compositions of the rectal swab samples were further analyzed by LEfSe. The LefSe analysis showed that *Burkholderia_Caballeronia_Paraburkholderia, Lachnospiraceae_NK4A136_group, Rhodococcus* and *Micrococcus* were enriched in the control group at day 14 of gestation; only *Collinsella* was enriched in the MSFAs group at day 14 of gestation; *Weissella, Trueperella, Desulfovibrio* and *Veillonella* were enriched in the control group at day 28 of gestation; and *Arcanobacterium* and *Candidatus_Saccharimonas* were enriched in the MSFAs group at day 28 of gestation (Figure 5). Thus, dietary supplementation with medium- and short-chain fatty acids in sows during early gestation had no significant effect on the diversity of rectal swab microbes in the sows, and only some microbes at the genus level showed significant differences.

### 3.6. Effects of Dietary Supplementation with MSFAs on Vaginal Microbial Diversity and Composition of Sows at 14 and 28 Days of Gestation

**Figure 6:**
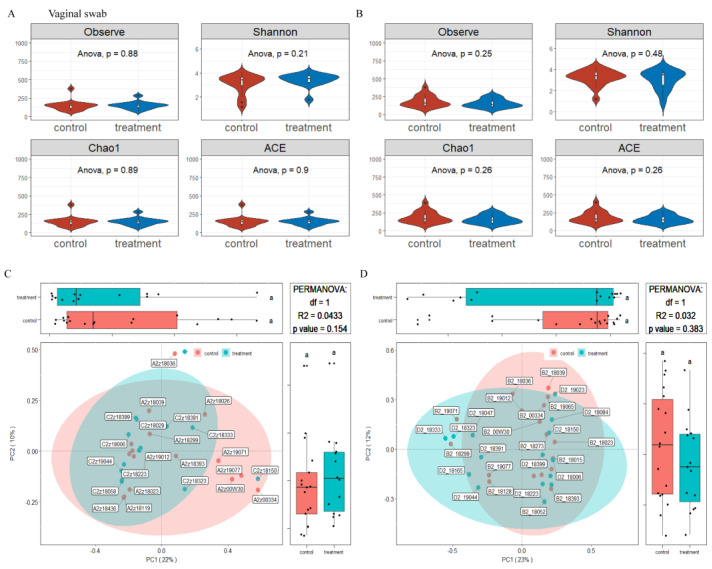
Effects of MSFAs during gestation on the vagina microbiota diversity of sows. Comparison of the number of observed species and Shannon, Chao1 and ACE indexes between the control and treatment groups at day 14 (**A**) and day 28 (**B**) of gestation. Beta diversity of the fecal microbiota in sows between the control and treatment groups at day 14 (**C**) and day 28 (**D**) of gestation based on weighted UniFrac distance. Data are expressed as the mean ± SEM (n = 11). Day 14 of gestation n: control = 12, treatment = 13, Day 8 of gestation n: control = 18, treatment = 15.

The microbiota diversity and compositions of vaginal samples in sows at 14 and 28 days of gestation were determined in the same way as in rectal swab samples. As showed in Figure 6, the alpha diversity values were no effect in dietary supplementation with MSFAs (*p* > 0.05). PCoA clustering revealed that the vaginal microbiota of sows was not significantly dispersed in the control and treatment groups (*p* > 0.05). Similarly, the addition of MSFAs to the diet also did not effectively change the vaginal microbiota diversity of sows at 14 or 28 days of gestation.

**Figure 7:**
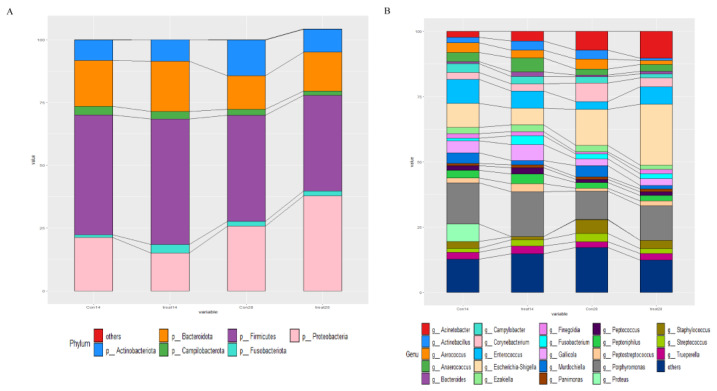
Effects of MSFAs during gestation on vaginal microbiota composition of sows. The relative abundances of different phyla (**A**) and genera (**B**) in the vaginal microbiota of control and treatment group sows at day 14 and day 28 at gestation.

**Figure 8:**
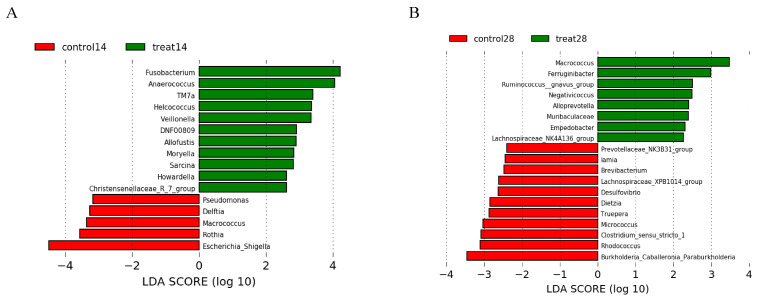
Effects of dietary supplementation with MSFAs on vaginal microbial diversity and composition of sows at 14 and 28 days of gestation. Linear discriminant analysis coupled with effect size (Lefse) of day 14 (**A**) and day 28 (**B**) vaginal microbiota composition of control and treated sows. Control14 (28), day 14 (28) of gestation and fed the basal diet. Treat14 (28), day 14 (28) of gestation and fed a basal diet supplemented with 0.1 SB, 0.05 SC and 0.1% SL.

The relative abundances at the phylum and genus levels in vaginal samples of all group across different days are shown in Figure 7. The top six phyla were *Firmicutes*, *Proteobacteria*, *Bacteroidota*, *Actinobacteriota*, *Campylobacterota* and *Fusobacteriota* (Figure 7A). These six phyla accounted for more than 99% of the reads for all sows. In addition, the top six genera in the gut microbiota of sows were *Porphyromonas, Escherichia-Shigella, Acinetobacter, Enterococcus, Gallicola* and *Corynebacterium* (Figure 7B). The microbial compositions of the rectal swab samples were further analyzed by LEfSe. The results showed that *Pseudomonas, Delftia, Macrococcus, Rothia* and *Escherichia-Shigella* were enriched in the control group at day 14 of gestation; *Fusobacterium, Anaerococcus, TM7a, Helcococcus, Veillonella, DNF00809, Allofustis, Moryella, Sarcina* and *Christensenellaceae_R_7_group* were enriched in the MSFA group at day 14 of gestation; *Prevotellaceae_NK3B31_group, Iamia, Brevibacterium, Lachnospiraceae_XPB1014_group, Desulfovibrio, Dietzia, Truepera, Micrococcus, Clostridum_sensu_stricto_1, Rhodococcus* and *Burkholderia_Caballeronia_Paraburknolderia* were enriched in the control group at day 28 of gestation; and *Macrococcus, Ferruginibacter, Ruminococcus_gnavus_group, Negativicoccus, Alloprevotella, Muribaculaceae, Empedobacter* and *Lachnospiraceae_NK4A136_group* were enriched in the MSFA group at day 28 of gestation (Figure 8). Similarly, dietary supplementation with medium- and short-chain fatty acids in sows during early gestation had no significant effect on the diversity of the vaginal microbes of sows, although there were more differences in genus-level microbes between the control and MSFA groups at day 14 or 28 of gestation.

## 4. Discussion

Early embryo loss is one of the key factors limiting successful reproduction in mammals [33,34]. Optimal hormone levels and implantation conditions, strongly influenced by maternal nutritional status, can effectively reduce early embryo loss. Supplementation with individual short- or medium-chain fatty acids is reported to improve maternal hormone levels and energy status [23,24,25,35,36]. However, it is still unknown whether the proportional combined use of these short- and medium-chain fatty acids achieves better effects. In the present study, our results showed that short- and medium-chain fatty acid supply strengthened steroidogenesis and luteinization processes in porcine granulosa cells by enhancing the expression of critical genes involved in these processes, including *Cyp19a1*, *Star*, *Pparγ*, *Fshr* and *Lhr*. In addition, short- and medium-chain fatty acids enhanced the receptivity of porcine endometrial epithelial cells. Furthermore, dietary supplementation with short- and medium-chain fatty acids improved the estrogen level of sows in early pregnancy, improved the reproductive performance of sows and provided a good intestinal and vaginal microbial environment.

Maintaining ovarian steroidogenesis is an important event for ovarian function and successful pregnancy. Research has shown that estrogens are important to primordial follicle formation and maintaining follicular development, through stimulating the final stage of ovulation and granulosa cell proliferation, promoting follicle-stimulating hormone-stimulated gene expression and even affecting the function and structure of female reproductive tissue [37,38,39,40]. Our previous study showed that short-chain fatty acids such as sodium butyrate and sodium caprylate can affect ovarian steroidogenesis and granulosa cell luteinization in rats [25]. In this study, we detected the effects of different concentrations of SB, SC and SL on PGCs cell activity by CCK-8 kit and selected the most appropriate concentration for subsequent tests (Figure S1). Although the actual physiological concentration of these fatty acids in follicles is much lower than our optimal concentration, the optimal concentration of these fatty acids did not negatively affect cell activity in vitro. Our data indicated that treatment with sodium butyrate or sodium caprylate could elevate the expression of *Cyp19a1*, which is a critical gene for estrogen synthesis and the prevention of cell apoptosis, in porcine granulosa cells [41]. In addition, it has been shown that both FSH and LH strongly stimulate *Cyp19a1* mRNA expression and estradiol and progesterone production in most mammalian mature granulosa cells by binding to the specific receptors *Fshr* and *Lhr*, respectively [42,43,44,45]. However, the impact of the combined use of short- and medium-chain fatty acids on the expression levels of *Lhr* and *Fshr* genes in granulosa cells has not been reported. Herein, our data showed that the combined use of short- and medium-chain fatty acids increased the mRNA level of the *Lhr* gene more effectively than sodium butyrate or sodium caprylate treatment, while sodium laurate had no effect on the expression of these genes. In addition, the combined use of short- and medium-chain fatty acids significantly increased the supernatant estrogen levels in porcine granulosa cells, while sodium butyrate or sodium caprylate treatment did not have such an effect, suggesting that the joint use of these fatty acids can more effectively promote estrogen secretion in porcine granulosa cells. Furthermore, our in vivo data indicated that dietary supplementation with MSFAs could significantly increase the serum estrogen level of sows during early pregnancy, indicating that the combined use of short- and medium-chain fatty acids could indeed strengthen ovarian estrogen secretion in sows during early pregnancy.

Embryo implantation is another decisive event for early embryo survival. During embryo implantation, approximately two thirds of implantation failures are related to poor uterine receptivity, while only one third of these failures are related to the embryo itself [46,47]. The biomarkers of uterine receptivity include *Hoxa10*, *Lif*, *αvβ3* integrin and *Egf*. *Hoxa10* is a homeobox gene required for endometrial receptivity to blastocyst implantation. *Hoxa10* has been reported as important to the endometrium by regulating endometrial stromal cell proliferation and epithelial cell morphogenesis [48]. In addition, it has been demonstrated that *Hoxa10* is related to pinopod development and is a morphological marker of high endometrial receptivity [49,50,51]. Blocking *Hoxa10* expression significantly reduces the number of endometrial pinopods. It has also been reported that the development of endometrial pinopods is associated with the increased expression of *Lif* and its receptors [52] and of *ανβ3* integrin [53]. In fact, the expression of *αvβ3* integrin in the human endometrium can be regulated by *Egf* [54]. Moreover, *Egf* is a potent inducer of *Lif* expression [55]. Our data demonstrated that sodium butyrate treatment could significantly increase the mRNA abundance of the *ανβ3* and *Lif* genes, while sodium caprylate treatment could increase the expression levels of *Egf* and *Hoxa10*. Sodium laurate only promotes the expression of *Hoxa10*. In contrast, the combined use of short- and medium-chain fatty acids significantly enhanced the endometrial expression levels of *Hoxa10*, *ανβ3*, *Lif*, and *Egf*. These results indicated that each fatty acid had different impacts on the biomarkers of endometrial receptivity. More importantly, the combined use of short- and medium-chain fatty acids was much more efficient in improving endometrial receptivity. These beneficial effects of the combined use of short- and medium-chain fatty acids were confirmed to effectively improve the embryo adhesion efficiency in vitro, and these beneficial effects were not due to differences in fat content but due to the specific types of fatty acid. Our previous data demonstrated that butyrate supplementation in the pregnant diet could not enhance the citrate cycle, and maintained the ATP levels and AMPK signaling, indicating that this level of butyrate supplementation could not influence the energy metabolism [56]. Our previous data also indicated that dietary butyrate supplementation during pregnancy primarily acted to promote the ovary function [25]. Therefore, we speculated that these functional fatty acids promoted the reproductive performance through enhancing the ovary function and uterine receptivity by not directly acting as the energy enhancing.

Notably, in addition to the improvement in ovarian steroidogenesis and endometrial receptivity, the combined use of short- and medium-chain fatty acids markedly inhibited specific pathogenic bacteria in the feces and vagina of sows during early pregnancy. It is well known that the gut and vaginal microbiomes during pregnancy have an impact on reproductive performance [57,58]. Gut microbiota imbalance can lead to pregnancy complications and adverse pregnancy outcomes, as well as polycystic ovary syndrome and endometriosis [58]. The vaginal microbiome is a key regulator of local inflammatory and immune pathways throughout pregnancy [59,60,61]. Studies have shown that dietary supplementation with sodium butyrate can promote intestinal health by modulating the microbial community in mice, piglets and poultry [62,63,64] and can also help to inhibit colitis and endometritis in mice [62,65,66]. Similarly, dietary supplementation with caprylic acid can significantly inhibit colonization by pathogenic bacteria (e.g., *Salmonella enteritidis* [67] and *Campylobacter* [68]) in the intestine of poultry. Moreover, caprylic acid can be used to treat monilial vaginitis [69]. In particular, lauric acid has been reported to have strong antibacterial activity, such as *Staphylococcus aureus*, *Streptococcus mutans*, *S. pyogenes*, *Escherichia coli*, and *H. pylori* [70,71]. In this study, the combined use of short- and medium-chain fatty acids in diets did not affect the diversity of intestinal and vaginal microorganisms in sows during early gestation; however, it significantly decreased, at the genus level, the rectal swab abundance of *Trueperella* and vaginal abundance of *Escherichia-Shigella* and *Clostridium_sensu_stricto_1* in sows. Numerous studies have reported that two species of *Trueperella, Trueperella pyogenes* and *Trueperella abortisuis*, are closely associated with vaginitis and abortion. *Trueperella pyogenes* is a well-known pathogen in domestic ruminants and pigs that causes mastitis and a variety of pyogenic infections [72]. Recently, *Trueperella pyogenes* was reported to be significantly associated with metritis in postpartum cows [73]. The high abundance of the *Trueperella abortisuis* strain seems to be responsible for abortion in farms [74]. In addition, the abundances of *Escherichia-Shigella* and *Clostridium_sensu_stricto_1* are relatively high in endometriotic sows [75]. Therefore, it is speculated that the combined use of short- and medium-chain fatty acids in diets protects against gut and vaginal pathogenic bacteria in sows during early pregnancy.

## 5. Conclusions

In conclusion, we found that the combined use of short- and medium-chain fatty acids improved the reproductive performance of sows associated with strengthening steroidogenesis and luteinization in ovarian granulosa cells and improving endometrial cell receptivity, while the treatment significantly decreased several specific pathogenic bacteria in the feces and vagina of sows during early pregnancy. These findings imply that the combined use of short- and medium-chain fatty acids has great potential to improve pregnancy outcomes in humans and other mammals.

## Figures and Tables

**Table 1:** Primers (pig) used for qPCR.

Genes	Forward (5′–3′)	Reverse (5′–3′)	Product Size (bp)
*C* *yp* *11* *a* *1*	GAGCAGGGAGAGTAGCAGTG	ACCAGGAGAGGGGATCTCAC	196
*Cyp19a1*	CCACATGGAAACCACCCATCT	TGCACAAACTCTTGGCCTCC	180
*Star*	CTGGAAGTCCCTCAAAGACCAA	GGGCTGAGCTTTAACACCTGG	177
*Pparγ*	TAGATGACAGCGACCTGGCGA	AGCAGCTTAGCAAAGAGCTGG	172
*Fshr*	CTCGGTTCCTTATGTGTAATC	TCAATGGCATAGTTGTGGTA	115
*Lhr*	ACCTGCCAACAAAAGAGCAG	GTCTCCTTGCTGTGCTTTCAC	81
*Egf*	GGATTTGCCCTGACCCTACT	TCTCTGTGCTGACATCGCTC	199
*Hoxa10*	TTTCACTTGTCCCGCTCTCC	CGCCTTTGGAATTGCCTTGA	178
*ανβ3*	CTGTTTGCCCATGTTTGGCT	TCCAGCCAATCTTCTCGTCA	167
*Lif*	CGCCCTCTTTATTCTCTACTACACA	TCACAGCACCAGGATTGAGG	215
*β-actin*	TGCGGGACATCAAGGAGAAG	AGTTGAAGGTGGTCTCGTGG	217

**Table 2:** Composition and Nutrient Composition of the Basal Diet for Sows (As-Fed Basis).

Items	Estrus Phase	Pregnancy Phase
Ingredients (%)		
Corn	61.94	61.71
Soybean meal	15.50	17.50
Wheat bran	-	8.10
Puffed soybeans	5.00	-
Beet meal	6.30	8.00
Fish meal	3.00	-
Glucose	3.00	-
Soybean oil	1.30	0.80
Dicalcium phosphate	1.53	1.31
Limestone	0.82	1.18
Vitamin-mineral premix ^1^	0.50	0.50
Salt	0.40	0.35
L-Lysine HCl	0.29	0.27
Choline Chloride (50%)	0.15	0.15
L-Threonine (98.5%)	0.11	0.09
L-Valine	0.09	0.02
DL-Methionine	0.05	0.02
L-Tryptophan	0.02	-
Dry matter (%)	88.06	88.09
Digestible energy(kcal/kg)	3355.00	3084.00
Metabolizable energy(kcal/kg)	3149.00	2920.00
Crude protein (%)	16.51	12.50
Ether extract (%)	5.26	3.83
Crude fiber (%)	3.50	4.78
Calcium (%)	0.90	0.85
Available phosphorus (%)	0.47	0.38
Digestible lysine (%)	0.95	0.62
Digestible methionine (%)	0.30	0.20
Digestible arginine (%)	0.92	0.63

^1^ Each kilogram of diet provides the following nutrients: zinc, 60 mg; iron, 95 mg; copper, 10 mg; iodine, 0.35 mg; selenium, 0.3 mg; manganese, 80 mg; vitamin A, 12,000 IU; vitamin D3, 2750 IU; vitamin E, 30 IU; vitamin K3, 2 mg; vitamin B12, 12 μg; vitamin B2, 6 mg; niacin, 40 mg; pantothenic acid, 12 mg; vitamin B6, 3 mg; biotin, 0.2 mg.

**Table 3:** Effects of medium- and short-chain fatty acid combination on body condition of sows ^1,2^.

Items	Control	Treatment	*p* Value
Number of sows	26	26	-
Parity	3.19 ± 0.19	2.85 ± 0.13	0.14
Weight after weaning (kg)	238.65 ± 4.66	240.92 ± 4.15	0.72
Weight at breeding (kg)	233.31 ± 4.78	236.46 ± 4.03	0.62
Weight at day 28 of gestation (kg)	233.46 ± 4.19	235.96 ± 3.35	0.65
Backfat thickness at breeding (mm)	14.77 ± 0.46	15.08 ± 0.52	0.67
Backfat thickness at day 28 of gestation (mm)	15.19 ± 0.55	16.12 ± 0.58	0.27

^1^ Values are mean ± SEM. ^2^ Significant differences at *p* ≤ 0.05.

**Table 4:** Effects of medium- and short-chain fatty acids on the reproductive performance of sows ^1,2^.

Items	Control	Treatment	*p* Value
Number of sows, n	26	26	--
Total piglets born per litter, n/litter	14.6 ± 0.45	15.6 ± 0.51	0.08
Total piglets born alive per litter, n/litter	13.2 ± 0.35	14.6 ± 0.45	0.009
Litter birth weight of all piglets born alive (kg)	18.1 ± 0.64	19.3 ± 0.63	0.09
Weight of piglets alive (kg)	1.38 ± 0.04	1.34 ± 0.04	0.25
Male alive piglets, n	7.08 ± 0.29	7.62 ± 0.29	0.20
Female alive piglets, n	6.19 ± 0.28	6.73 ± 0.32	0.22
Weak, n	1.69 ± 0.26	1.77 ± 0.30	0.43
Stillborn, n	1.27 ± 0.28	0.96 ± 0.17	0.18
Deformed, n	0.12 ± 0.06	0.12 ± 0.06	0.5
Mummfied, n	0.15 ± 0.12	0.04 ± 0.04	0.18

^1^ Values are mean ± SEM. ^2^ Significant differences at *p* ≤ 0.05.

**Table 5:** Effects of medium- and short-chain fatty acids on reproductive performance of sows ^1,2^.

Items	Control	Treatment	*p* Value
Estradiol (pg/mL)	42.60 ± 6.68	84.20 ± 14.00	0.04
Progesterone (pg/mL)	748.00 ± 76.60	654.00 ± 98.50	0.52
Total cholesterol (mmol/L)	1.47 ± 0.06	1.32 ± 0.08	0.21
HDL (mmol/L)	0.64 ± 0.02	0.56 ± 0.04	0.17
LDL (mmol/L)	0.74 ± 0.04	0.67 ± 0.05	0.33

^1^ Values are mean ± SEM. ^2^ Significant differences at *p* ≤ 0.05.

## Data Availability

The data supporting the reported results and conclusions can be found in the submitted figure and tables. Additional research materials and protocols that are relevant to the study are available from the corresponding author upon reasonable request.

## Supplementary Materials

The following supporting information can be downloaded at: https://www.mdpi.com/article/10.3390/nu14204405/s1, Figure S1: CCK-8 assay of PGCs treated with different concentration of SB (A), SC (B), SL (C) for 24 h; Figure S2: Fatty acid composition in serum of sows at 28 days of gestation under different treatments.; Table S1: Dietary information for diets with fat supplementation.
