# Supplementary material for: The Combined Use of Medium- and Short-Chain Fatty Acids Improves the Pregnancy Outcomes of Sows by Enhancing Ovarian Steroidogenesis and Endometrial Receptivity
Source: Nutrients. 2022 Oct 20;14(20):4405. doi: 10.3390/nu14204405 (PMC9607977; doi:10.3390/nu14204405)
Supplement: Supplementary file 1 [file nutrients-14-04405-s001.zip › nutrients-1905334-supplementary.pdf]

**Supplementary data**

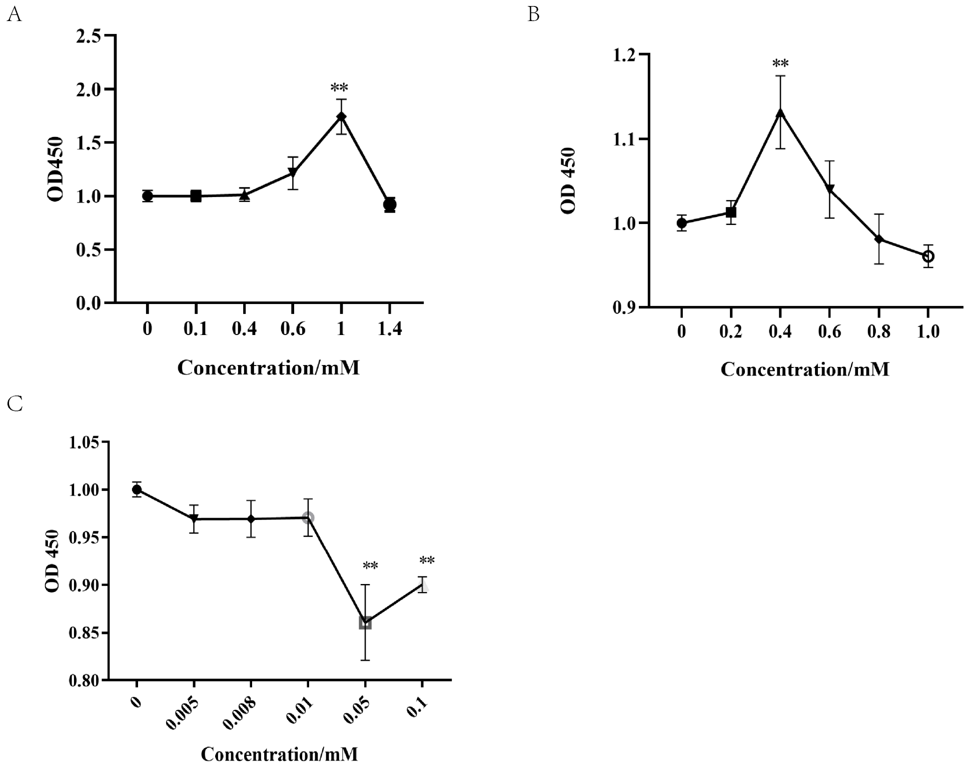


**Figure S1.** CCK-8 assay of PGCs treated with different concentration of SB (A), SC (B), SL (C) for 24 h. The results are presented as the means ± SEM. n=6. Different letters between bars indicate ** P ⩽ 0.01 by one-way ANOVA followed by post hoc Tukey's tests. ns, nonsignificant (P ＞ 0.05). SB, sodium butyrate; SC, sodium caprylate; SL, sodium laurate; MSFAs, SB + SC + SL.

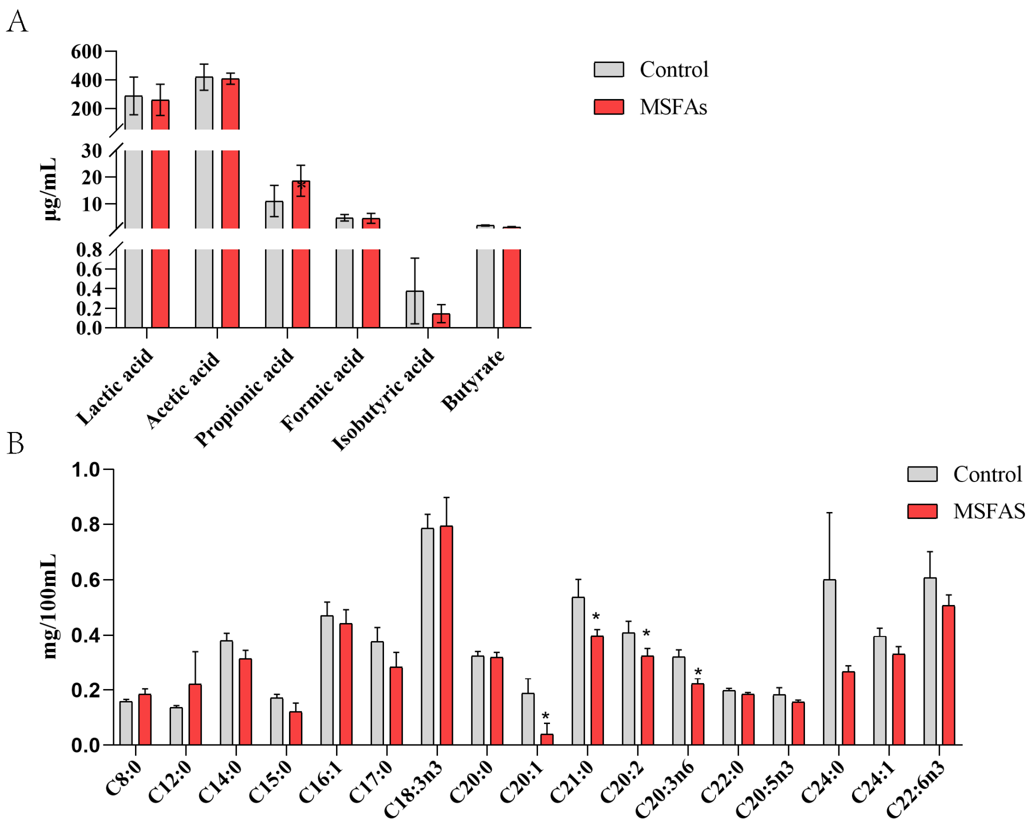


**Figure S2.** Fatty acid composition in serum of sows at 28 days of gestation under different treatments. (A) Volatile fatty acid composition in serum of sows. (B) Medium and long chain fatty acid composition in serum of sows. The results are presented as the means ± SEM. n=5. Different letters between bars indicate * P ⩽ 0.05 by one-way ANOVA followed by post hoc Tukey's tests. ns, nonsignificant (P ＞ 0.05).

**Table S1. Dietary information for diets with fat supplementation.**

| Items | Soybean oil, % | SB, % | SL, % | SC, % |
|---|---|---|---|---|
| **Control_Estrus phase** | 1.30 | 0.00 | 0.00 | 0.00 |
| **MSFAs_Estrus phase** | 1.30 | 0.10 | 0.05 | 0.10 |
| **Control_Pregnancy phase** | 0.80 | 0.00 | 0.00 | 0.00 |
| **MSFAs_Pregnancy phase** | 0.80 | 0.10 | 0.05 | 0.10 |
